# COVID-CT-MD, COVID-19 computed tomography scan dataset applicable in machine learning and deep learning

**DOI:** 10.1038/s41597-021-00900-3

**Published:** 2021-04-29

**Authors:** Parnian Afshar, Shahin Heidarian, Nastaran Enshaei, Farnoosh Naderkhani, Moezedin Javad Rafiee, Anastasia Oikonomou, Faranak Babaki Fard, Kaveh Samimi, Konstantinos N. Plataniotis, Arash Mohammadi

**Affiliations:** 1 Concordia Institute for Information Systems Engineering (CIISE), Concordia University, Montreal, Canada; 2 Department of Electrical and Computer Engineering, Concordia University, Montreal, QC Canada; 3 Department of Medicine and Diagnostic Radiology, McGill University Health Center-Research Institute, Montreal, QC Canada; 4 Department of Medical Imaging, Sunnybrook Health Sciences Centre, University of Toronto, Toronto, Canada; 5 Faculty of Medicine, University of Montreal, Montreal, QC Canada; 6 Department of Radiology, Iran university of medical science, Tehran, Iran; 7 Department of Electrical and Computer Engineering, University of Toronto, Toronto, Canada

**Keywords:** Viral infection, Imaging

## Abstract

Novel Coronavirus (COVID-19) has drastically overwhelmed more than 200 countries affecting millions and claiming almost 2 million lives, since its emergence in late 2019. This highly contagious disease can easily spread, and if not controlled in a timely fashion, can rapidly incapacitate healthcare systems. The current standard diagnosis method, the Reverse Transcription Polymerase Chain Reaction (RT- PCR), is time consuming, and subject to low sensitivity. Chest Radiograph (CXR), the first imaging modality to be used, is readily available and gives immediate results. However, it has notoriously lower sensitivity than Computed Tomography (CT), which can be used efficiently to complement other diagnostic methods. This paper introduces a new COVID-19 CT scan dataset, referred to as COVID-CT-MD, consisting of not only COVID-19 cases, but also healthy and participants infected by Community Acquired Pneumonia (CAP). COVID-CT-MD dataset, which is accompanied with lobe-level, slice-level and patient-level labels, has the potential to facilitate the COVID-19 research, in particular COVID-CT-MD can assist in development of advanced Machine Learning (ML) and Deep Neural Network (DNN) based solutions.

## Abstract (summary)

**Table:** 

Measurement(s)	Low Dose Computed Tomography of the Chest • viral infectious disease
Technology Type(s)	digital curation • image processing technique
Factor Type(s)	sex • gender • age group • weight • clinical characteristics • covid-19 RT-PCR result • follow-up data
Sample Characteristic - Organism	Homo sapiens

Machine-accessible metadata file describing the reported data: 10.6084/m9.figshare.13583015

## Background & Summary

Since its first emergence in late 2019, novel Coronavirus (COVID-19) has drastically changed the world, impacting several aspects of the modern life. According to the World Health Organization (WHO), as of January 2021, more than 200 countries have confirmed positive COVID-19 cases, leading to more than 90 million cases and almost 2 million reported fatalities. Considering statistics and impacts together with the fact that COVID-19 can easily spread if infected cases are not isolated/treated in a timely fashion, sensitive and accessible diagnosis systems are of significant importance. Reverse Transcription Polymerase Chain Reaction (RT- PCR)^1^, which is currently considered as the gold standard diagnosis technique, suffers from relatively low sensitivity and the outcome highly depends on the area from which the sample is obtained, and therefore, it is operator dependant. More importantly, this test is time consuming that is not desirable as time is a critical factor in isolating, treating, and preventing the transition of COVID-19. Medical imaging is highly topical and potentially of significant clinical importance as the pandemic evolves, especially where access to RT-PCR tests is limited, unreliable or where the timeframe for an RT-PCR test might provide less optimal care than an immediate confirmation. Being able to identify COVID-19-related respiratory complications, Chest Radiographs (CXR), can play an important complementary role for the RT- PCR test to asses complications. Moreover, imaging follows more standardized protocols and is less dependent on the operator’s experience. COVID-19 manifestation in images has shown correlation with the disease severity, providing a means for its progression assessment. The outcome of the RT-PCR, however, does not identify the disease severity or stage. Although, CXR can act as a quantitative method to assess the extent of COVID-19 involvement and estimate the risk of Intensive Care Unit (ICU) admission, it still has lower sensitivity compared to Computed Tomography (CT)^2^. Due to high sensitivity and rapid access, chest CT plays a significant role in diagnosis and management of COVID-19 and has been recognized as the most sensitive imaging modality to detect complications^3^. It is worth noting that the developed imaging-based AI algorithms for the purpose of COVID-19 diagnosis can pave the path for the development of similar automatic systems for potential future pandemics, for which RT-PCR tests are not available.

Despite the high potential of CT in contributing to the COVID-19 research and clinical usage, publicly available datasets are mostly limited to a few number of cases, are not accompanied with other types of respiratory diseases to facilitate comparisons, and are not associated with suitable labels. Furthermore, cases may be collected from different sources with different imaging protocols, limiting a unified study. In a few identified datasets, available CT scans are limited to only infected slices, rather than the complete volume. Another important aspect that should be considered in the available datasets is that whether labels are available in a patient-level, slice-level, and lobe-level fashion. The later can further contribute to identify the location of the COVID-19 infection. Finally, different types of labels and information, suitable for different tasks, are provided in identified datasets. Table 1 provides an overview of the available datasets along with the provided COVID-19 related information.

**Table 1:** Available COVID-19 CT scan datasets. NA stands for not available.

Dataset	Number of cases			Label type		Data Source		CT volume		Label Level		
	COVID	CAP	Normal	Classification	Segmentation	Multiple	Single	Available	Not available	Patient-level	Slice-level	Lobe-level
Reference^20^	49	NA	NA		✓	✓		✓			✓	
Reference^21^	20	NA	NA		✓	✓		✓			✓	
Reference^22^	20	NA	NA		✓	✓		✓			✓	
Reference^23^	856	NA	254	✓		✓		✓		✓		
Reference^24^	216	NA	55	✓		✓			✓		✓	
Reference^25^	60	NA	60	✓		✓			✓		✓	
Reference^4^	95	NA	282	✓			✓	✓		✓	✓	
Reference^26^	2,980	NA	NA	✓		✓		✓		✓		
COVID-CT-MD	169	60	76	✓			✓	✓		✓	✓	✓

The introduced COVID-19 CT scan dataset, referred to as the COVID-CT-MD, is applicable in Machine Learning (ML) and deep learning studies of COVID-19 classification. In particular, COVID-CT-MD dataset consists of 169 confirmed positive COVID-19 cases (gathered from 2020/02/23 to 2020/04/21), 76 normal cases (gathered from 2019/01/21 to 2020/05/29), and 60 Community Acquired Pneumonia (CAP) cases (gathered from 2018/04/03 to 2019/11/24). All these cases are collected from Babak Imaging Center in Tehran, Iran, and labeled by three experienced radiologists in patient-level, slice-level, and lobe-level manners. Patient-level label refers to a single diagnosis assigned to the participant, whereas slice-level and lobe-level refer to identifying slices and lobes demonstrating infection, respectively. More importantly, the whole CT volume is available for all the participants. COVID-CT-MD is presented in Table 1, along with the previous datasets, to highlight its differences. Regarding Reference^4^, we would like to mention that while this Reference provides only COVID-19 and normal cases, COVID-CT-MD provides CAP cases additionally. Furthermore, COVID-CT-MD is the only classification-related dataset that contains lobe-level information, which can significantly improve and contribute to the localization and analysis of the COVID-19 infection.

## Methods

This section provides a description of the data collection procedure, inclusion criteria, and de-identification. Furthermore, detailed statistics of the data is presented to facilitate its usage. More importantly, applicability of the COVID-CT-MD dataset for development of ML/DNN solutions is explained. This section is concluded by describing the possible limitations of the provided dataset. This research work is performed based on the policy certification number 30013394 of Ethical acceptability for secondary use of medical data approved by Concordia University, Montreal, Canada. Furthermore, informed consent is obtained from all the patients.

### Data collection

The COVID-CT-MD dataset contains volumetric chest CT scans of 169 patients positive for COVID-19 infection, 60 patients with CAP, and 76 normal patients. COVID-19 cases are collected from February 2020 to April 2020, whereas CAP cases and normal cases are collected from April 2018 to December 2019 and January 2019 to May 2020, respectively, in Babak Imaging Center, Tehran, Iran. Three main criteria are considered by three radiologists for classifying the participants, as follows:

1. Imaging findings including:
  Ground Glass Opacities (GGOs), referring to hazy transparent opacities;
  Consolidation pattern, which means the air in the alveoli and peripheral bronchioles is replaced by fluid;
  Crazy Paving, referring to thickened interlobular septa and intralobular lines superimposed on a background of ground-glass opacity;
  Bilateral and multifocal lung involvement;
  Peripheral distribution; and
  More distribution in lower lobes.
2. Clinical findings including symptoms, characteristics, patient history, and RT-PCR outcome if available; and
3. Epidemiology, referring to whether the participant comes from high risk areas or has had close contact with a positive COVID-19 patient.

If a participant is identified positive according to all three criteria, COVID-19 label is assigned. Otherwise, the participant is classified as either CAP or normal. This procedure is followed by the three radiologists. Subsequently the majority voting is adopted for the final assignment. The three radiologists have 88.9% agreement in identifying COVID-19, CAP, and normal cases, whereas the first and second radiologists have 91.1% agreement, the first and third radiologists have 97.4% agreement, and the second and third radiologists have 89.1% agreement.

A subset of 54 COVID-19, and 25 CAP cases were analyzed by the first radiologist to identify and label slices with evidence of infection. The labeled subset of the data contains 4,957 number of slices demonstrating infection and 18,392 number of slices without infection.

Besides CT slices, clinical data is collected for the patients, which includes the following:

- Patients’ age;
- Patients’ gender;
- Patients’ weight;
- Clinical characteristics: including symptoms, reason for scanning, and patients’ history;
- Surgery history;
- Follow-up: some of the COVID-19 patients are followed-up after scanning and their status including recovery, hospital admission, and death is recorded;
- RT-PCR: positive RT-PCR outcome is available for some of the COVID-19 patients.

CT scans are comprised of cross-sectional 2D images from thin sections of the body (slices), creating a 3D representation of the structures inside the body. In the modern CT scanners, a rotating X-ray generator sends multiple X-ray beams into the object from multiple angles. The amount of the radiation passed through the object is then captured by sensitive radiation detectors, followed by a computer-assisted process, which reconstructs the information obtained from the detectors into detailed sequential images using image reconstruction techniques^5^. All images in COVID-CT-MD are obtained from a SIEMENS, SOMATOM Scope scanner in the axial view, using the helical acquisition technique, i.e., the patient is moved through the gantry while the X-ray beams and detectors are spinning rapidly around the patient. The images are reconstructed using the Filtered Back Projection (FBP) reconstruction method^6^. The reconstruction matrix size (output size of the images) is set to 512 × 512, and the D40s reconstruction kernel is used to reduce the blurring and noise by modifying the frequency contents of the data during the image reconstruction in the scanner^7^. Finally, all images are provided in the Hounsfield Unit and saved in the Digital Imaging and Communications in Medicine (DICOM) format. It is worth mentioning that following the recommended chest CT protocols for suspected cases or follow up of metastasis, bronchiectasis, interstitial lung disease and pulmonary infections^8^ all images are Non-Contrast CT (NCCT) and none of them is CT Pulmonary Angiography (CTPA). Acquired images are, consequently, reconstructed into high resolution CT (HRCT).

**Fig. 1:**
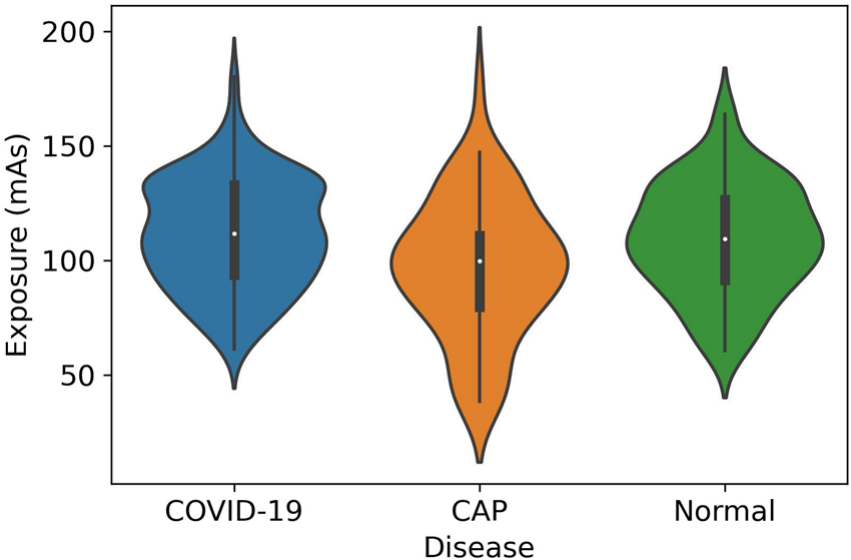
The distribution of the Exposure values for COVID-19, CAP and Normal cases.

Table 2 shows different CT acquisition settings, where Peak KiloVoltage (kVp) and Exposure Time affect the radiation exposure dose, while slice thickness represents the axial resolution. As shown in Table 2, slice thickness, kVP, and exposure time are almost the same with a few variations in a few CAP cases. Distance of Source to detector and Distance of Source to patient, which are traditionally referred to as SID and SOD, respectively, are also the same in all cases except for a few CAP cases. The minimum and maximum exposure value (in mAs) used in the scanning process is also presented in Table 2. The exposure value determines the total radiation dose in CT scan. The distribution of the exposure values is illustrated by the violin plots for each disease type in Fig. 1. Accordingly, the mean and standard deviation of the exposure values are reported in Table 3.

**Table 2:** CT scan settings used to acquire the COVID-CT-MD dataset.

Diagnosis	Slice Thickness (mm)	Peak Kilovoltage (kVp)	Exposure Time (ms)	X-ray Tube Current (mA)	SID (mm)	SOD (mm)	Exposure values (mAs)
**COVID-19**	2	110–130	600	153–343	940	535	61.2–180.0
**CAP**	2	110–120	420–600	94–500	940–1040	535–570	38.4–175.24
**Normal**	2	110	600	132–343	940	535	60.4–163.71

**Table 3:** The statistical parameters (mean and standard deviation) of the Exposure values.

Diagnosis	Exposure mean	Exposure standard deviation
**COVID-19**	111.43	23.70
**CAP**	96.64	29.75
**Normal**	109.18	23.97

#### CT Acquisition care in the medical imaging department

As COVID-19 is highly contagious, all the staff of the medical imaging department involved in the CT acquisition are provided with personnel protective equipment (PPE). More importantly, there is a minimum of 5-minute time slack between two consecutive CT scans, allowing enough time to sanitize the CT scanner.

### Data inclusion and exclusion criteria

All cases with confirmed clinical diagnosis are included in the dataset. Nevertheless, during the data collection procedure, there were some cases related to the late 2019, with manifestations similar to those of COVID-19. However, as the first COVID-19 case in Iran is reported in early February 2020, these cases were excluded from the dataset. Furthermore, according to the radiologists’ assessment, images with poor quality and visible artifacts were excluded. In summary, 320 cases were initially screened, among which 5% (15 cases) were excluded according to the radiologists’ judgement, allowing 305 high quality CT studies.

### De-identification

To respect the patients’ privacy and comply with the DICOM supplement 142 (Clinical Trial De-identification Profiles)^9^, we have de-identified all the CT studies by removing or obfuscating every names, UIDs, dates, times, comments, and center-related information. Some helpful DICOM attributes related to the patients’ gender and age, the scanner type, and the image acquisition settings have been retained to preserve the statistical characteristics of the dataset. Patient’s ID and UID attributes which are necessary to retain the consistency of the CT studies are replaced by new generated values which does not allow the identification of the patients.

### Data statistics

**Fig. 2:**
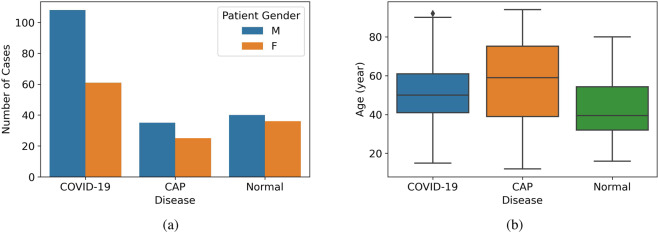
(**a**) The number of cases separated by the patient’s gender. (**b**) The distribution of age for COVID-19, CAP and Normal cases.

The demographic distribution of the dataset describing the gender and age distributions is illustrated in Table 4 and Fig. 2. Please note that, no restrictions were imposed on the participants to indicate a binary response. As shown in Fig. 2(a), males outnumbered females in this dataset. However, we would like to mention that although male cases are dominant, according to a recent study^10^, there is no correlation between the CT score and participants’ gender. Furthermore, this dominance is common in most of the COVID-19-related datasets^3^, possibly because men are more vulnerable to COVID-19, compared to women^11^. The boxplot in Fig. 2(b) represents the important statistical parameters of the patients’ age distribution. As shown in this boxplot, normal cases are mainly distributed in lower ages, while CAP cases are distributed in a wide range of ages with a higher average age. Regarding the ethnicity of the patients, the participants are Iranian (more than 60% Persian). Potential combination of the COVID-CT-MD dataset with other available ones, presented in Table 1, improves the applicability of AI algorithms to different populations.

**Table 4:** Gender and age distribution in COVID-CT-MD.

Diagnosis	Cases	Gender	Age (year)
**COVID-19**	169	108 M/61 F	51.96 ± 14.39
**CAP**	60	35 M/25 F	57.7 ± 21.7
**Normal**	76	40 M/36 F	43.4 ± 14.1

**Fig. 3:**
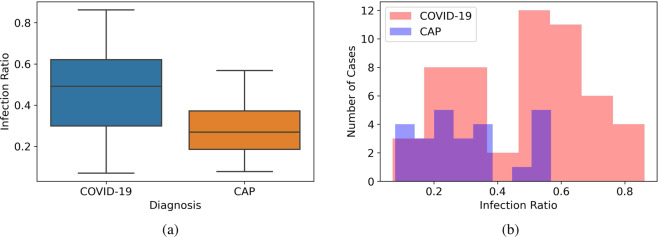
(**a**) The distribution of the Infection Ratio in the labeled dataset for COVID-19 and CAP cases. (**b**) The histogram of the Infection Ratio in the labeled dataset for COVID-19 and CAP cases.

As previously stated, part of the dataset is analyzed and the slice-level labels are extracted. The number of labeled cases and slices demonstrating infection are presented in Table 5. Infection ratio in this table represents the ratio of the slices demonstrating infection to the total number of slices in a CT scan, which varies for different cases based on the severity and stage of the disease. The minimum and maximum values for the infection ratio in the labeled dataset are presented in Table 5. The distribution of the Infection Ratio is also illustrated by the boxplots in Fig. 3(a), which demonstrate a higher infection ratio in COVID-19 cases compared to CAP cases. The histogram of the Infection Ratio values is illustrated in Fig. 3(b).

**Table 5:** The number of cases, Slices, and Infection Ratio in the labeled dataset.

Diagnosis	Cases	Slices Demonstrating Infection	Slice without infection	Infection Ratio
**COVID-19**	54	3779	4269	7.0%–86.2%
**CAP**	25	1178	2718	7.8%–56.8%

**Fig. 4:**
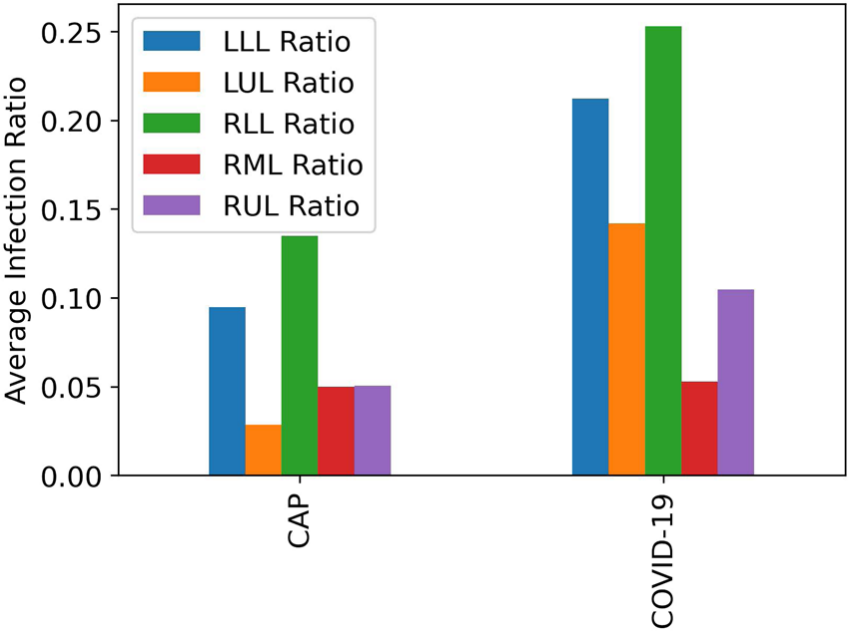
Average Infection Ratio in each lobe of the lung for COVID-19 and CAP cases in the labeled dataset.

In addition to the described slice-level labels, the detailed distribution of infection in each lobe of the lung is provided by the radiologists. Table 6 indicates the number of cases and slices with infection demonstrated in specific lung regions. Similar to Fig. 3, where the infection ratio was presented for the total slices with infection in the lung, the average of lobe infection ratios are presented in Fig. 4, illustrating the average ratio of slices demonstrating infection in a particular lobe to the total number of slices in a CT scan. As evident in Table 6 and Fig. 4, the average infection ratio in the lower lobes is higher in both COVID-19 and CAP cases compared to other lung regions in our labeled dataset.

**Table 6:** Number of cases and slices, respectively, demonstrating infection in each lobe.

Diagnosis	LLL	LUL	RLL	RML	RUL
**COVID-19**	42&1669	38&1120	45&2008	26&420	29&826
**CAP**	13&374	5&117	18&519	7&186	9&208
**Total**	56&2079	43&1237	63&2527	33&606	38&1034

LLL: Left Lower Lobe–LUL: Left Upper Lobe–RLL: Right Lower Lobe and Lingula–RML: Right Middle Lobe–RUL: Right Upper Lobe.

### Limitations

Although all cases and labels are confirmed by three experienced radiologists, we would like to describe a few limitations that the data users may encounter. These limitations are as follows:

- The slice and lobe labeling processes focus more on regions with distinctive manifestations rather than minimal findings.
- Not all the COVID-19 patients have confirmed positive RT-PCR result, as this test was not publicly accessible in Iran at the time of the first emergence of the COVID-19. Furthermore, the high load of patients in need of COVID-19 examination, did not allow for an inclusive RT-PCR test. The diagnosis of some patients in the COVID-CT-MD dataset is confirmed based on the CT findings, as well as the clinical results and epidemiology.
- Although most of the cases with low quality CT scans are excluded, there may still be some cases with mild motion artifact which is inevitable, since COVID-19 patients suffer from dyspnea.
- During the slice and lobe labeling process, some suspicious areas adjacent to the chest wall and diaphragm are not labeled as “infected”, due to their poor distinction.

## Data Records

**Fig. 5:**
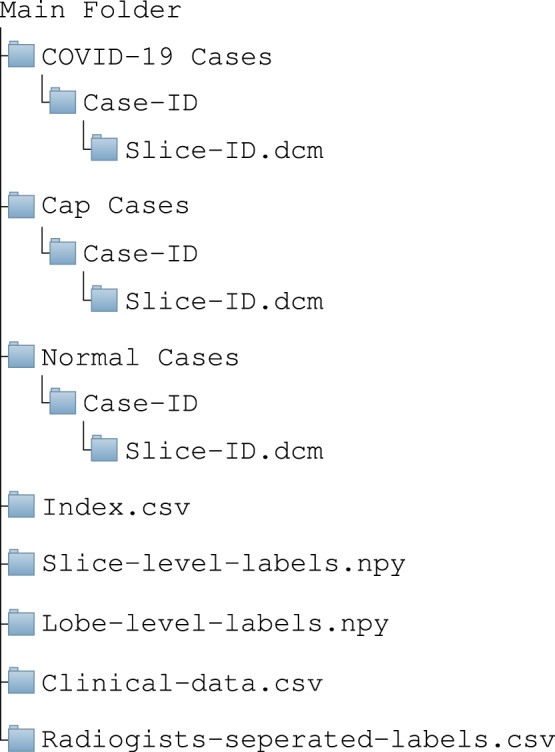
Structure of the data included in COVID-CT-MD dataset.

The diagram in Fig. 5 shows the structure of the COVID-CT-MD dataset. The COVID-CT-MD dataset is accessible through Figshare^12^. COVID-19, CAP and Normal participants are placed in separate folders, within which patients are arranged in folders, followed by CT scan slices in DICOM format. “Index.csv” is related to the patients having slice-level and lobe-level labels. The indices given to patients in “Index.csv” file are then used in “Slice-level-labels.npy” and “Lobe-level-labels.npy” to indicate the slice and lobe labels. “Slice-level-labels.npy” is a 2D binary Numpy array in which the existence of infection in a specific slice is indicated by 1 and the lack of infection is shown by 0. In “Slice-level-labels.npy”, the first dimension represents the case index and the second one represents the slice numbers. “Lobe-level-labels.npy” is a 3D binary Numpy array in which the existence of infection in a specific lobe and slice is determined by 1 in the corresponding element of the array. Like the slice-level array, in “Lobe-level-labels.npy”, the two first dimensions represent the case index and slice numbers respectively. The third dimension shows the lobe indices which are specified as follows:

- 0: Left Lower Lobe (LLL)
- 1: Left Upper Lobe (LUL)
- 2: Right Lower Lobe (RLL)
- 3: Right Middle Lobe (RML)
- 4: Right Upper Lobe (RUL)

It is worth noting that CT slices are sorted based on the “Slice Location” value stored in the corresponding DICOM tag “(0020,1041) - DS - Slice Location”. The slice-level and lobe-level labels are provided according to described slice order. The researchers, however, can re-arrange the slices using other CT attributes based on their preference, as long as they re-arrange the labels accordingly. The COVID-CT-MD dataset is also accompanied with the clinical data, stored in “Clinical-data.csv”. Finally, to facilitate the inter-observer reliability studies, labels assigned by the three radiologists are separately provided in “Radiologists-separated-labels.csv”.

## Technical Validation

Two noteworthy parameters in the studies using CT scans are the quality control and calibration of the scanning device. The longest time period between the scanner auto-calibration and the study in the COVID-CT-MD dataset is 1 day, which ensures calibrated and accurate performance of the scanning device. Furthermore, there is an annual SIEMENS quality control that ensures the absence of ring artifacts in the acquired CT scans.

## Usage Notes

With the increasing number of COVID-19 patients, healthcare workers are overwhelmed with a heavy workload, lowering their concentration for a proper diagnosis. Accurate and timely COVID-19 diagnosis, on the other hand, is a critical factor in preventing the disease transition, treatment, and resource allocation. Machine Learning (ML), in particular Deep Learning (DL) based on Deep Neural Networks (DNN), is shown to be practical and effective in COVID-19 diagnosis and severity assessment. The COVID-CT-MD dataset is specifically designed to facilitate application of ML/DL in COVID-19-related tasks. In particular, this dataset can be used towards:

- A patient-level binary classification^13,14^ to distinguish COVID-19 from all other cases.
- A patient-level multi-class classification^13^ to identify COVID-19, CAP, and normal participants.
- A slice-level^15^ and lobe-level classification to separate infected slices and lobes from non-infected ones for further analysis.
- Slice-level and lobe-level labels can be used as additional inputs to segmentation models^16^, to focus on only infected slices.
- Slice-level and lobe-level labels can be used in generative models to generate artificial COVID-19 images, towards increasing the security of the healthcare systems and developing attack resilient solutions^17^.

We have utilized the COVID-CT-MD dataset in our recent studies^18,19^, to classify participants as COVID-19 or non-COVID (Normal and CAP). The models proposed in these studies consist of two stages. In the first stage, infected slices (COVID-19 and CAP) are separated from healthy ones, through a developed Capsule Network. Consequently, in the second stage, infected slices are used to classify patients as COVID-19 or non-COVID. While the first stage exploits the provided slice-level labels, the patient-level ones are used in the second stage. It is worth mentioning that we continue the slice/lobe labeling to include all the cases. Although the slice/lobe labels are still incomplete, the first stage models in the underlying studies^18,19^ achieve an accuracy of almost 93%. Data users are encouraged to train and test their methods on the COVID-CT-MD dataset and compare their results, accordingly.

## Data Availability

The Python code used to generate the statistical analysis and plots is shared within the same Figshare link^12^, with the name “Statistical_Analysis.py”.
